# Supplementary material for: Circulating Endocannabinoids in Canine Cutaneous Mast Cell Tumor
Source: Animals (Basel). 2024 Oct 16;14(20):2986. doi: 10.3390/ani14202986 (PMC11503820; doi:10.3390/ani14202986)
Supplement: Supplementary file 1 [file animals-14-02986-s001.zip › animals-3242560-supplementary.pdf]

Supplementary Table 1: Control group of healthy dog

#	Breed	Sex	Age (years)	Bodyweight (Kg)	BCS
1	Cocker	M	11	17	5
2	Lagotto	NM	3	15	5
3	Cross breed	F	6	10	5
4	Dogo Argentino	F	2	36	5
5	Cross breed	NM	5	30	5
6	Poodle	SF	7	12	5
7	American Bully	M	4	27	5
8	Cross breed	NM	5	12	5
9	Cross breed	NM	5	39	5
10	Cross breed	M	6	11	5
11	Cross breed	SF	7	35	5

Supplementary Table 2: Comparative analysis of circulating endocannabinoid levels between dogs in clinical stages I and II versus stage III; dogs that received antihistaminic treatment versus those that did not; dogs with tumor sizes greater than 3 cm versus less than 3 cm; and dogs with ulceration versus those without ulceration.

Factor	Endocannabinoid	Group	Mean	SEM	P-Value	95% CI Lower	95% CI Upper
Clinical Stage	AEA	I+II	5.8	0.92	0.872	-3.54	4.14
		III	5.5	1.85			
	2-AG	I+II	139.08	23.9	0.888	-85.74	74.12
		III	144.89	33.25			
	PEA	I+II	29.67	1.41	0.665	-6.95	4.52
		III	30.89	2.73			
	OEA	I+II	18.72	2.4	0.92	-9.51	8.61
		III	19.17	4.13			
Antihistaminic	AEA	Yes	5.77	0.98	0.89	-4.17	3.5
		No	6.11	1.14			
	2-AG	Yes	142.09	50.02	0.966	-108.28	103.75
		No	144.35	23.34			
	PEA	Yes	28.53	1.82	0.554	-8.35	3.87
		No	30.77	1.75			
	OEA	Yes	21.58	5.43	0.589	-8.43	14.95
		No	18.32	2.62			
Tumor Size	AEA	> 3 cm	6.21	1.63	0.705	-3.03	4.51
		< 3 cm	5.48	1.03			
	2-AG	> 3 cm	130.85	31.48	0.736	-94.77	65.65
		< 3 cm	145.41	23.92			
	PEA	> 3 cm	32.63	3.59	0.213	-2.91	10.07
		< 3 cm	29.05	1.05			
	OEA	> 3 cm	23.24	5.39	0.106	-2.56	12.04
		< 3 cm	17.06	1.8			

Ulceration	AEA	Yes	4.12	1.25	0.243	-5.63	1.17
		No	6.35	1.06			
	2-AG	Yes	180.58	40.35	0.165	-125.64	9.31
		No	230.43	35.2			
	PEA	Yes	20.57	3.1	0.611	-8.25	5.26
		No	25.33	2.65			
	OEA	Yes	15.24	4.12	0.49	-7.6	14.07
		No	18.99	3.48			
